# Hypereosinophilic Syndrome, Cardiomyopathies, and Sudden Cardiac Death in Superinvasive Opisthorchiasis

**DOI:** 10.1155/2019/4836948

**Published:** 2019-05-09

**Authors:** Vitaly G. Bychkov, Vladimir M. Zolotukhin, Elena D. Khadieva, Svetlana V. Kulikova, Ivan M. Petrov, Svetlana G. Berdinskih, Semen D. Lazarev, Lola F. Morozova, Evgeny N. Morozov

**Affiliations:** ^1^Tyumen State Medical University, Tyumen, Russia; ^2^Pathology Bureau, Clinical Center “Medical City”, Tyumen, Russia; ^3^Khanty-Mansiysk State Medical Academy, Khanty-Mansiysk, Russia; ^4^Tyumen Cardiology Research Center, Tomsk National Research Medical Center, Tyumen, Russia; ^5^Sechenov University, Moscow, Russia; ^6^Russian Medical Academy of Continuing Professional Education, Moscow, Russia

## Abstract

Cardiovascular pathology in patients with superinvasive opisthorchiasis is characterized by severe changes in haemodynamics and myocardial metabolism, impaired automatism, excitability, and conduction of the heart muscle. An analysis of 578 cases (medical and outpatient records and reports of pathoanatomical and forensic autopsies) recorded in healthcare facilities treating opisthorchiasis patients with a hyperendemic focus was carried out. We identified a set of cardiac changes in patients with hypereosinophilic syndrome associated with superinvasive opisthorchiasis infection, classified the pathological processes in accordance with ICD-10, and described their pathogenesis.

## 1. Introduction

Cardiovascular pathology in patients with superinvasive opisthorchiasis (SO) is characterized by severe changes in haemodynamics and myocardial metabolism, impaired automatism, excitability, and conduction of the heart muscle. Significant, direct correlations between pain syndrome incidence and the duration of invasion, the frequency of re-infections, and stress fluctuations of the host have been found [1–3].

Hypereosinophilia is a pathological condition resulting from hypereosinophilic syndrome (HES), in which the number of eosinophilic leukocytes in peripheral blood is greater than 15% (1.5 × 10^9^/L) [3–5]. Hypereosinophilic syndrome (as an episode) is observed in all patients with opisthorchiasis with multiple re-infections and an invasion period >15 years [3].

The most detailed description of the myocardial state in different types of HES in SO was achieved by physicians, who revealed the development of severe forms of myocarditis and described cases of sudden cardiac death (SCD) in patients with SO-associated myocarditis [6, 7]. In a study of opisthorchiasis patients with a hyperendemic focus (Khanty-Mansiysk Autonomous District), the prevalence of nonviolent, sudden death in people with noncoronarogenic heart disease increased. The mechanisms of cardiac reconstruction in SO have been studied insufficiently [8]; therefore, an understanding of cardiovascular pathology in opisthorchiasis is still urgent and requires further study.

Currently, HES as the focus of opisthorchiasis is less common; however, HES as a complication of SO is underrepresented in the literature. There is a need to look for other risk factors for SCD and to develop measures for the primary prevention of cardiac death in patients with tissue helminthiases.

## 2. Materials and Methods

**Figure 1:**
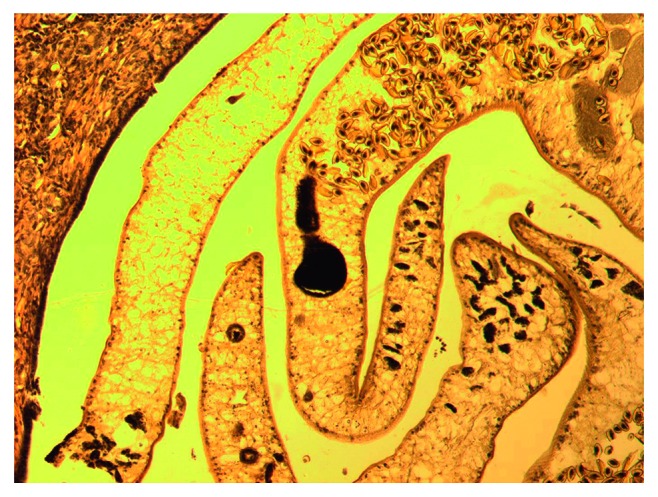
Superinvasive opisthorchiasis (SO). *Opisthorchis felineus* in the different stages of the ontogenesis by haematoxylin and eosin (HE) staining (magnification 400x).

**Figure 2:**
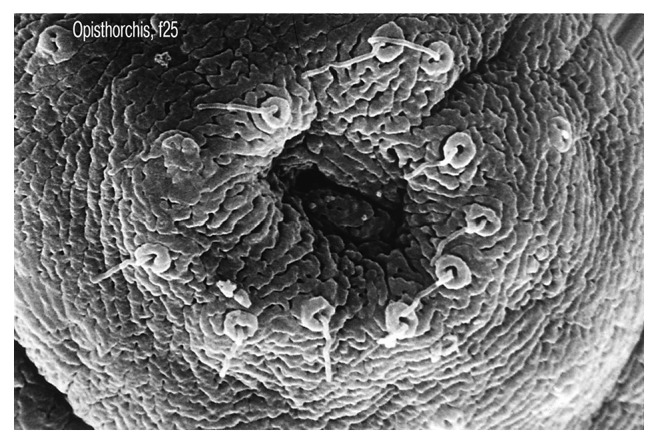
Oral sucker of *Opisthorchis felineus* by scanning electron microscopy.

An analysis of 578 cases (medical and outpatient records and reports of pathoanatomical and forensic autopsies) recorded in healthcare facilities treating opisthorchiasis patients with a hyperendemic focus was carried out. The average age of patients was 49.7 ± 3.4 years, and the average age of those who died was 52.3 ± 2.9 years. Males (*n*=217) and females (*n*=361) were included, with a female to male ratio of 1.6 : 1. A total of 110 hearts as well as livers (Figures 1 and 2) from deceased patients were subjected to morphological study, including hearts from those with sudden cardiac death (*n*=16). The preparations were stained with haematoxylin and eosin and van Gieson's stain using the Selye and Slinchenko methods. The degree of cellular infiltration and sclerotic processes was recorded by determining the index of the area occupied by cellular elements and fibrous structures (%), as well as their proportion in the composition of infiltrates. The stained preparations were subjected to light-optical analysis, and the images of histological preparations, taken with a microscope and Canon EOS 5D digital camera, were saved on a computer. Using the UTHSCSA Image Tool for Windows 3.0 program, the area of metabolic myocardial necrosis was determined. Mathematical and statistical processing of the data was performed on a personal computer using Statistica 6.1 software (Statsoft®).

## 3. Results and Discussion

Cardiac pain (52.42%), rhythm disturbance (72.31%), palpitation (28.20%), and expansion of cardiac borders to the left (44.29%) were the most common pathologies in patients with SO; ECG changes were detected in 26.47% of patients, and mitral valve prolapse was detected in 1/3 of the individuals examined. Microscopic examination of those who died from accidental causes and complications associated with SO revealed the following changes in heart membranes: fuchsinophilic dystrophy of cardiomyocytes and their apoptosis with no leukocyte environment around the dead elements and diffuse cardiosclerosis. These changes are attributed to the group of lesions associated with hypoxia due to Botkin syndrome, i.e., systematic, short vasospasm of the heart vessels.

**Figure 3:**
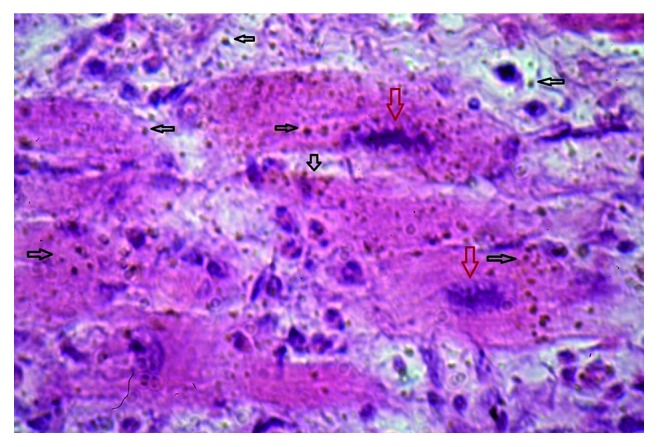
Eosinophilic myocarditis. Parasite's exometabolite dispersal is shown by black arrows and altered cardiomyocytes are shown by red arrows (haematoxylin and eosin (HE) staining, magnification 400x).

**Figure 4:**
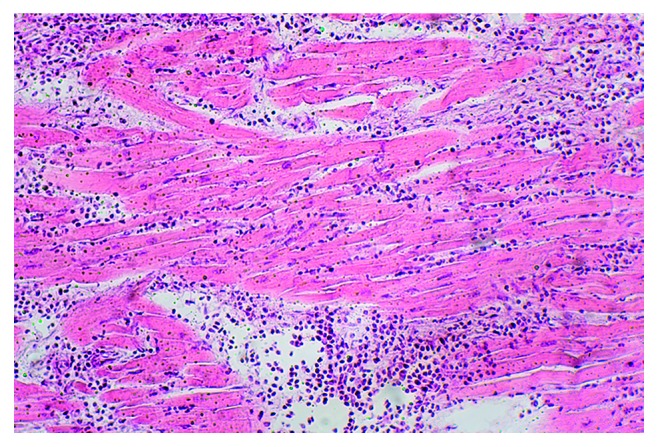
SCD, HES, and diffuse eosinophilic myocarditis were visualized with haematoxylin and eosin (HE) staining (magnification 100x).

**Figure 5:**
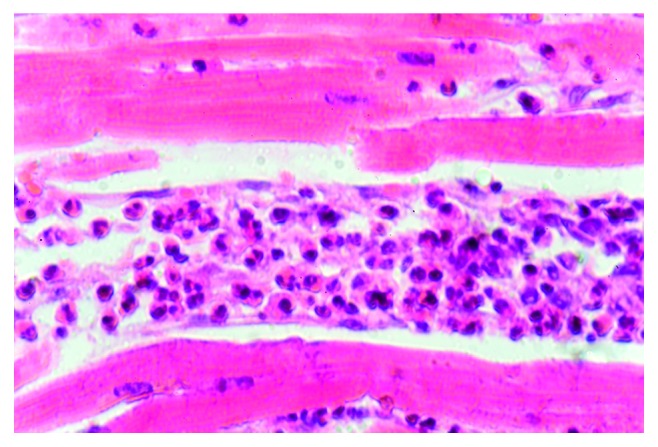
HES, diffuse eosinophilic myocarditis, intermuscular localization of granulocytes, dystrophy, and death of cardiomyocytes were visualized with haematoxylin and eosin (HE) staining (magnification 200x).

**Figure 6:**
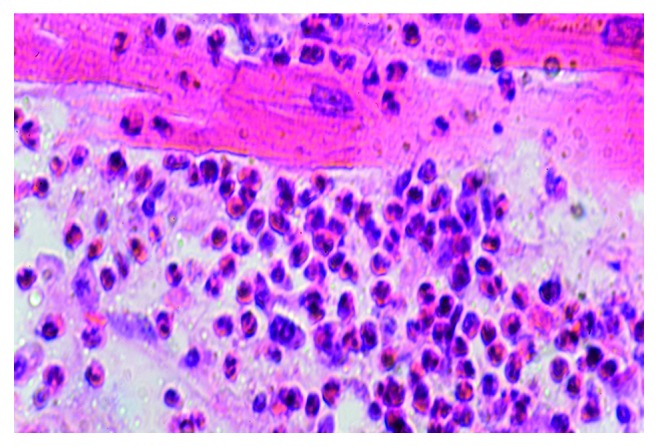
HES complicated by eosinophilic myocarditis, an aggression of granulocytes, and myolysis of cardiomyocytes were visualized with haematoxylin and eosin (HE) staining (magnification 200x).

**Figure 7:**
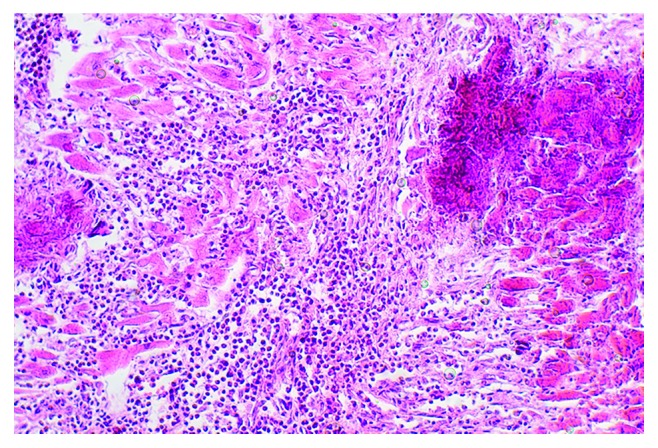
SCD, HES, and metabolic necrosis complicated by eosinophilic myocarditis were visualized with haematoxylin and eosin (HE) staining (magnification 200x).

The most pronounced pathology observed in eosinophilic myocarditis was the scattering and contamination of cardiomyocytes with opisthorchis exometabolites. Subsequently, an aggression of eosinophilic leukocytes followed by myocardial cell death led to the formation of extensive foci of deparenchymatization of the muscle membrane and perivascular, cystoid, and diffuse cardiosclerosis. The direction of collagen fibres coincided with the parasite's exometabolite dispersal (Figures 3–6). In some areas of the myocardium, there were foci of cell death induced by metabolic disorders (metabolic necrosis with no signs of inflammation) (Figure 7). The cellular composition of myocardial infiltrates in HES and sudden death associated with HES is presented in Table 1.

In the definitive stage of eosinophilic leukocyte aggression, fragments of nuclei, cytoplasm, and cytolemma were observed, suggesting the “suicide” of eosinophils during contact with antigens (*O. felineus* metabolites and contaminated cardiomyocytes), which is denoted as EETs (eosinophil extracellular traps). The mechanism is similar to that of NETs (neutrophil extracellular traps), in which leukocytes (neutrophilic and/or eosinophilic) release a meshwork of DNA fibres and granule proteins.

Statistical processing of the data on infiltrate cellular composition showed a significant increase in leukocytes and a decrease in endotheliocytes (angiogenesis depression), fibroblasts, and fibrocytes (lack of motivation to substitute damaged myocardial sites) in SCD associated with HES patients compared to that in those who died by violence in combination with HES (*p* < 0.01 − 0.001). Moreover, in HES, with the development of sudden cardiac death, a significant increase in the area of metabolic myocardial necrosis was observed (*p* < 0.001).

Sudden cardiac death (SCD) is a topical health issue in Russia and abroad. The most frequent (more than 80%) cause of sudden cardiac death is coronary heart disease, while ventricular fibrillation (including reperfusion) [9] plays a key role in the mechanism of thanatogenesis [5, 10]. Cardiomyopathies cause sudden cardiac death in 8.5% of cases, and 71.4% of these cases are alcohol-related cardiomyopathies [10]. In a population of opisthorchiasis patients with a hyperendemic foci, coronarogenic sudden cardiac death is less common; however, cardiomyopathies (inflammatory and noninflammatory) caused by HES, complicated by eosinophilic myocarditis, account for 19%, according to our data, as well as for over 17% according to other authors [11].

Thus, pathological changes in the heart in SO are explained by coronary blood supply disturbances of a reflex type similar to those of Botkin syndrome. These changes were accompanied by paroxysmal cardialgia, resulting in diffuse cardiosclerosis. Cholecystocoronary syndrome was most prominent in patients with various forms of cholecystitis (chronic SO complication).

**Figure 8:**
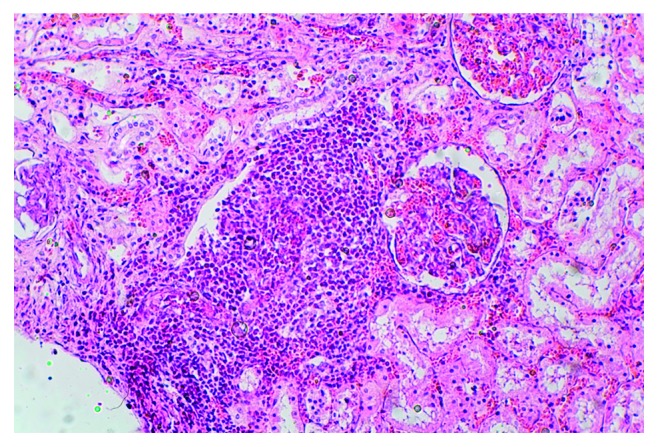
HES and eosinophilic infiltrates in the kidneys were visualized with haematoxylin and eosin (HE) staining (magnification 200x).

**Figure 9:**
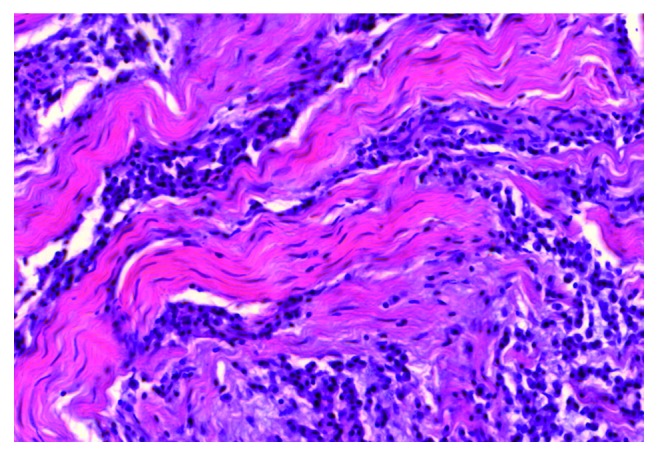
SCD, HES, and diffuse eosinophilic infiltrates in the gums were visualized with haematoxylin and eosin (HE) staining (magnification 200x).

**Figure 10:**
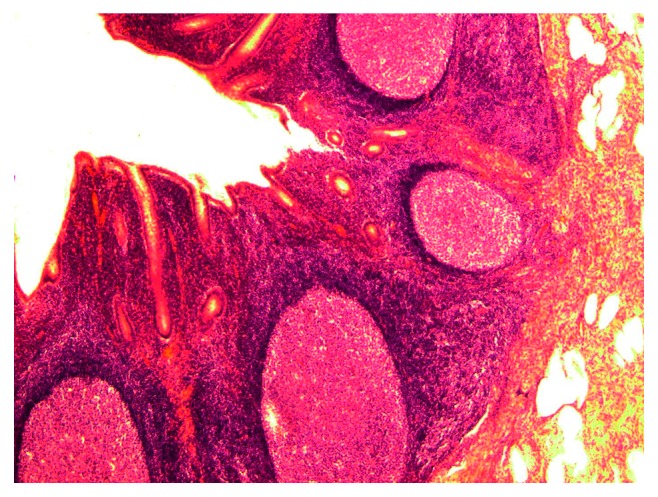
HES and hyperplasia of lymphoid cells in the appendix were visualized with haematoxylin and eosin (HE) staining (magnification 200x).

Eosinophilic infiltrates in HES are consistently found in the kidneys (Figure 8), gums (Figure 9), and mucosae of the small and large intestines. In addition, pronounced hyperplasia of lymphoid cells with the expansion of reactive foci of lymphoid follicles is observed along the entire length of the entodermal canal (Figure 10). Depositions of *O. felineus* exometabolites are found in all organs, including the intestines, lungs, skin, and gums. Clinical signs of HES depend on the localization (target organs) of eosinophilic leukocytes and *O. felineus* metabolites; thus, they can either manifest as general, nonspecific reactions, or, for example, if the appendix is affected, the symptoms of acute appendicitis are evident, while infiltration in the kidneys is accompanied only by pain and does not result in organ insufficiency. Thanatogenetic mechanisms of sudden cardiac death in HES involve the development of ventricular fibrillation. Another mechanism of SCD, thromboembolic syndrome due to a parietal thrombus in the setting of eosinophilic pancarditis, was observed in one case.

Hypereosinophilic syndrome in SO is caused by opisthorchis exometabolites that contaminate cardiomyocytes; the latter subsequently becomes autoantigens and targets for eosinophilic leukocytes. In addition to the cellular immune response mentioned above, there is a humoural component (antibodies to myocardium *α*-myosin) [12].

Blood hypereosinophilia in opisthorchiasis correlates with tissue hypereosinophilia. A high level of IL-5 and hyperproduction of IgE and IgG are involved in the mechanisms of hypereosinophilia [12].

Prevention of SO complications involves the exclusion of primary and recurrent infections, elimination of chronic inflammation, and/or surgeries in cases of gallbladder inflammation. These measures, along with cholecystectomy, also prevent choleperitonitis. Antiparasitic therapy plays a key role in the prevention of heart pathologies and sudden cardiac death. Dehelminthization should be conducted during the chronic phase of the disease. Antiparasitic drugs used in the acute phase and at the peak of HES are complicated by increased eosinophilia, including tissue infiltration, the exacerbation of eosinophilic myocarditis, and vasculitis, thus aggravating SCD. Blood tests and autopsy material analyses showed that even with eosinophilia in over 10% of all white blood cells, dehelminthization caused an increase in eosinophils by 123%–185%, with the development of tissue eosinophilia and eosinophilic myocarditis. Treatment of patients with HES (Loffler's syndrome), which is consistently encountered in SO, is rather challenging and requires an individualized approach.

## 4. Conclusion

In superinvasive opisthorchiasis, hypereosinophilic syndrome should be referred to as a symptomatic HES, in which eosinophilia refers to a reactive, nonclonal, unlimited impairment of target organs with an infectious parasitic agent. Heart diseases in superinvasive opisthorchiasis should be attributed to secondary cardiomyopathies characterized as dilated, noninflammatory (degeneration of cardiomyocytes, cardiosclerosis), and metabolic (metabolic necrosis). Inflammatory cardiomyopathies develop with HES and eosinophilic myocarditis, which, in turn, are induced by the contamination of cardiomyocytes by opisthorchis metabolites, turning them into target cells for antigens. Similar mechanisms for alteration, exudative, and proliferative processes in the heart are observed in other helminthic infections [12–14].

## Figures and Tables

**Table 1:** Cellular composition of myocardial infiltrates around (*O. felineus*) metabolites in hypereosinophilic syndrome and sudden cardiac death in patients with superinvasive opisthorchiasis.

No.	Components of infiltrates	Proportion of cells in the infiltrate, *M* ± *m* (%)	
		HES	SCD associated with HES
1	WBC	66.17 ± 5.14	88.73 ± 4.81
	Eosinophilic	76.31 ± 3.86	77.28 ± 2.84
	Lymphocytes	8.34 ± 3.44	7.10 ± 2.08
	Neutrophilic	10.63 ± 2.68	12.12 ± 3.16
	Monocytes	3.24 ± 0.83	3.00 ± 0.72
	Basophilic	1.48 ± 0.01	0.50 ± 0.06
2	Endotheliocytes	8.66 ± 2.68	3.14 ± 1.22
3	Fibroblasts	7.47 ± 3.01	3.05 ± 2.82
4	Fibrocytes	10.14 ± 1.94	0.84 ± 0.08
5	Macrophages	5.19 ± 2.63	4.08 ± 1.83
6	Plasmocytes	2.37 ± 0.94	0.16 ± 0.04

## Data Availability

All data underlying the findings described in the manuscript are fully available without restriction. All relevant data are within the manuscript.
